# Comprehensive treatment of opioid addiction in patients receiving opioid agonist maintenance therapy

**DOI:** 10.1192/j.eurpsy.2026.10877

**Published:** 2026-07-31

**Authors:** N. Halytska-Pasichnyk

**Affiliations:** 1 Psychiatry, Narcology, Medical psychology, Dnipro State Medical University; 2 Department of OAMT, “Dnipropetrovsk multiprofile clinical hospital for provision of psychiatric care” Dnipropetrovsk regional council”, Dnipro, Ukraine

## Abstract

**Introduction:**

Opioid **addiction** is a chronic relapsing disorder that poses a significant threat to public health worldwide. In modern medicine, opioid agonist maintenance therapy is the gold standard of treatment. Long-term therapy is superior to short-term detoxification, where relapse rates reach 90% within the first month.

**Objectives:**

Methadone therapy, as a form of opioid agonist substitution maintenance therapy, stabilizes patients by reducing cravings and withdrawal symptoms, but its effectiveness increases significantly when combined with psychotherapeutic interventions. Therefore, drug treatment should be integrated with psychotherapy to address the cognitive, emotional, and behavioral factors of addiction.

**Methods:**

A total of 150 patients aged 26 to 64 years participated in the study, including 117 (78.0%) men and 33 (22.0%) women. The participants were divided into the main group (101 individuals) and the comparison group (49 individuals). The study employed psychodiagnostic methods, including SCL-90-R, HAM-A, HAM-D, the Buss-Durkee Hostility Inventory, MoCA, and WHOQOL-BREF. In the comprehensive therapeutic process, in addition to methadone therapy, the observation group used psychocorrectional and psychotherapeutic interventions.

**Results:**

After treatment, the total sample shows a predominance of mild/absent symptoms, but the control group has a higher percentage of moderate/severe conditions (58% with moderate/severe/severe anxiety vs 2% in the main group). This indicates a potential advantage of complex therapy in the main group. Less social anxiety in the main group: 87% very low/low vs 71% in the control group. Less hostility in the main group: 96% very low/low vs 82% in the control group. Anxiety and depression (Hamilton scales): The main group has significantly better results: almost complete reduction to mild/absent states. Control — higher risk of residual symptoms. Cognitive deficit (MoCA): The main group — mostly absent (59%), control — mostly mild (82%). Better neuroprotection is possible in the main group. SCL-90 symptoms: General trend — the main group shifted to “very low/low” (85–96% on the scales), the control group — more average/high (up to 32%).

**Conclusions:**

The results of the study showed that the comprehensive treatment of opioid **addiction** based on methadone therapy is an evidence-based approach that combines pharmacological stabilization with psychotherapeutic support. Pharmacological therapy effectively retains patients and reduces risks, while psychotherapy provides long-term remission and social reintegration. For maximum effectiveness, individualized plans, monitoring and an interdisciplinary team are necessary. Comprehensive treatment involves the integration of MT with psychotherapeutic, social and medical services.

**Disclosure of Interest:**

None Declared

